# Evaluation of Exercise Tolerance in Dialysis Patients Performing Tai Chi Training: Preliminary Study

**DOI:** 10.1155/2016/5672580

**Published:** 2016-07-28

**Authors:** Wioletta Dziubek, Katarzyna Bulińska, Mariusz Kusztal, Joanna Kowalska, Łukasz Rogowski, Agnieszka Zembroń-Łacny, Tomasz Gołębiowski, Bartosz Ochmann, Weronika Pawlaczyk, Marian Klinger, Marek Woźniewski

**Affiliations:** ^1^Department of Physiotherapy, University of Physical Education, Paderewskiego 35 Street, 51-612 Wroclaw, Poland; ^2^Department and Clinic of Nephrology and Transplantation Medicine, Wroclaw Medical University, Borowska 213 Street, 50-556 Wroclaw, Poland; ^3^Non-Public Medical College of Wroclaw, Nowowiejska 69 Street, 50-340 Wroclaw, Poland; ^4^Faculty of Medicine and Health Sciences, University of Zielona Gora, Licealna 9 Street, 65-417 Zielona Gora, Poland; ^5^Department of Physical Education, University of Physical Education, Paderewskiego 35 Street, 51-612 Wroclaw, Poland

## Abstract

*Introduction*. Patients with end-stage renal disease (ESRD) have poor physical performance and exercise capacity due to frequent dialysis treatments. Tai Chi exercises can be very useful in the area of rehabilitation of people with ESRD. Objectives. The aim of the study was to assess exercise capacity in ESRD patients participating in 6-month Tai Chi training.* Patients and Methods*. Twenty dialysis patients from Wroclaw took part in the training; at the end of the project, 14 patients remained (age 69.2 ± 8.6 years). A 6-minute walk test (6MWT) and spiroergometry were performed at the beginning and after 6 months of training. Results. After 6 months of Tai Chi, significant improvements were recorded in mean distance in the 6MWT (387.89 versus 436.36 m), rate of perceived exertion (7.4 versus 4.7), and spiroergometry (8.71 versus 10.08 min).* Conclusions*. In the ESRD patients taking part in Tai Chi training, a definite improvement in exercise tolerance was recorded after the 6-month training. Tai Chi exercises conducted on days without dialysis can be an effective and interesting form of rehabilitation for patients, offering them a chance for a better quality of life and fewer falls and hospitalisations that are the result of it.

## 1. Introduction

Chronic kidney disease (CKD) is a common illness that affects approximately 150 million people worldwide each year. Furthermore, the number of patients with end-stage CKD, when it becomes necessary to start dialysis, is quickly growing. Currently, more than half of the patients included in the dialysis programmes in Europe are people over the age of 65. The largest increase among all patients who qualified for dialysis has been recorded in the 70+ age group. For example, in 2012 in Poland, 64% of all qualified subjects were over the age of 65.

An increase in morbidity is associated with the phenomenon of prolonging average life expectancy and the escalation of lifestyle diseases. Patients with CKD are burdened with a number of comorbidities, such as diabetes and cardiovascular diseases, which are often the cause of kidney failure and, at the same time (cardiovascular events), are responsible for their death. Poor physical performance predicts the high risk of mortality and cardiovascular events in patients with CKD.

Progressive renal failure is often accompanied by an unhealthy lifestyle, including a lack of regular physical activity. This contributes to a deterioration of functioning in everyday life. The culmination of poor functioning in end-stage renal failure occurs when a patient requires renal replacement therapy. It is estimated that, after each month of dialysis, the level of physical activity of people with end-stage renal disease (ESRD) decreases by 3.4% [1]. Compared to healthy subjects and patients with a milder form of the disease, the level of exercise tolerance, exercise capacity, strength, and endurance of those on dialysis are significantly reduced [2, 3]. It is estimated that the ability for physical exertion in patients with ESRD is 60–65% compared to healthy individuals with sedentary lifestyles [4, 5].

The introduction of physical training to a therapeutic process of people with CKD is recommended regardless of the severity of the disease. Recommendations of the Kidney Disease: Improving Global Outcomes (KDIGO) indicate a need to introduce rehabilitation programmes based on the principles of cardiac training due to the high risk of cardiovascular events [6]. Patients with ESRD are offered two possibilities of supervised physical training: during dialysis treatments (the first two hours or up until ultrafiltration at 2.5 l of fluids) and on days without dialysis.

There is a lot of scientific evidence confirming the effectiveness of regular physical exercise during and between dialysis treatments [7–11]. Physical training performed at least twice a week for 45–60 minutes on average has an effect, i.a., on improved aerobic capacity, reduced tissue insulin resistance, and blood pressure, thereby modifying cardiovascular risk factors [12]. Regular physical exercise also causes hypertrophy of skeletal muscles (an increase of surface area of type I and types IIa and IIx fibres in a cross section) [13], improving muscle strength and power, lowering fatigability, and causing an overall increase in physical functioning in patients with ESRD [11, 14]. An improvement in physical fitness significantly improves quality of life and daily functioning [15].

Physical exercises performed on days without dialysis offer more health benefits compared to training undertaken during dialysis treatments [16]. On such days, patients generally feel better; they can perform free-range movements at all levels and train in a standing position. An additional benefit of these types of activities is their group character, which improves social contact and motivation to exercise and maintains regularity. However, it requires constant supervision by a qualified physiotherapist and doctor during each training unit. Because of the burden of coexisting comorbidities, there are programmes introduced with low (30–49% of HR_max_; 25–44% VO_2max_) to moderate (50–69% HR_max_; 45–59% VO_2max_) intensity, which is gradually increased as patients adapt to the exercise [17].

Tai Chi exercises have been very useful in the area of rehabilitation of people with ESRD. Because of their low intensity, fluidity of movement, mindfulness, and diversity of forms, they are considered safe and have many benefits in terms of psychophysical aspects. The unquestionable advantage of Chinese gymnastics is its relaxing nature, together with breathing, overcoming the resistance of one's own body, carefully shifting the center of gravity, and normalising muscle tone, which are of great importance to people with ESRD due to myopathy, renal osteodystrophy, and concomitant cardiovascular disorders [18]. The low intensity of Tai Chi exercises is recommended for those leading a sedentary lifestyle, people who are frail, and the elderly [19].

In the literature, there is little scientific information about the effects of Tai Chi exercises on patients undergoing dialysis [20–23], including only two investigations concerning Tai Chi exercises on days without dialysis [21, 22]. Therefore, the aim of the study was to assess the exercise capacity of patients with end-stage renal failure participating in a six-month Tai Chi training conducted on days without dialysis.

## 2. Methods

Studies were performed in the Laboratory of Functional Studies at the Department of Physiotherapy of the University of Physical Education in Wroclaw. Tai Chi training was undertaken by twenty dialysis patients of the Clinic of Nephrology and Transplantation Medicine at the Wroclaw Medical University. All were residents of the city who met the following inclusion criteria: end-stage renal failure, hemodialysis therapy for at least six months prior to the commencement of research, attending practitioner's consent to participate in training, and adequate dialysis therapy (dialysis adequacy ratio *Kt*/*V* > 1.2: protein catabolic rate 0.8–1.4 g protein/kg of body weight/day). The final analysis excluded patients who died during the study, received a kidney transplant, did not want to complete the questionnaire again, or attended fewer than 36 training sessions (<75% of the training cycle). Lack of cooperation (especially due to dementia) and absence of written informed consent were other reasons for exclusion from the study.

**Figure 1:**
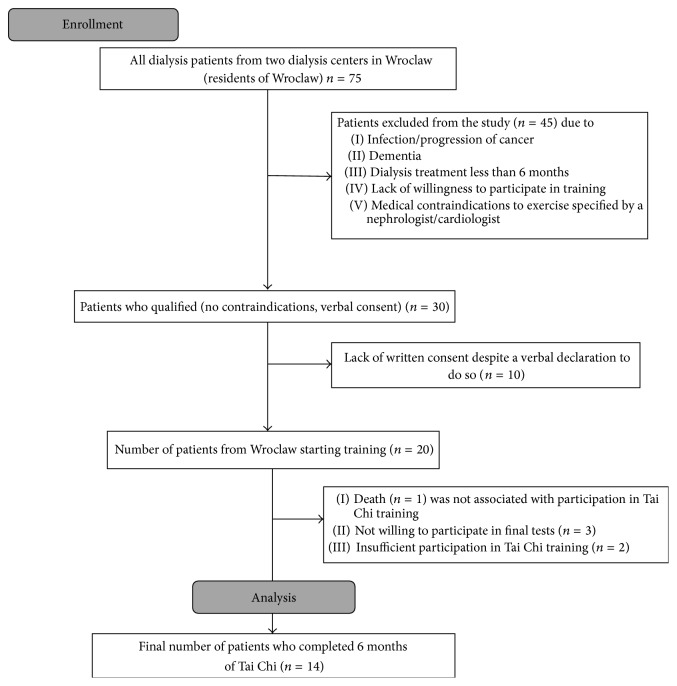
The recruitment process towards the study group.

To participate in the study, patients were qualified by a nephrologist and a cardiologist (see study flow chart, Figure 1). The nephrologist's permission to participate in the study was based on the patient's recent health status and biochemistry results. Each patient was informed of the voluntary nature of the study and of the option to withdraw from the exercise training. To carry out the study, approval from the Bioethics Committee of the University of Physical Education in Wroclaw was obtained. All patients gave their informed written consent to participate in the study. During the study project, one patient died of pneumonia (high CRP, WBC) and the death was not associated with participation in Tai Chi training.

### 2.1. Outcome Measures

A personal questionnaire, six-minute walk test (6MWT), and spiroergometry on a K4b2 by COSMED (Italy) ergospirometer were used in the study. Each patient filled out a personal questionnaire once, before the training. At the beginning (*t*_1_) and after six months of Tai Chi exercise training (*t*_2_), a 6MWT and spiroergometry were performed.

### 2.2. A Personal Questionnaire

The questionnaire consisted of two parts and included both sociodemographic data (age, gender, marital status, education, and number of years on dialysis) and clinical data related to the disease (date of diagnosis, date of commencement of dialysis, cause of renal failure, and number of comorbidities). The first part was to be filled by the patient and the other part by the staff based on the medical history report.

### 2.3. 6MWT

A 6MWT was performed in accordance with the guidelines of the American Thoracic Society, that is, along a 30 m corridor under medical supervision [24]. The respondent was asked to walk for six minutes at a pace that they used daily. The study measured the distance covered during the entire test. This test allowed for the assessment of the levels of exercise tolerance and exercise capacity. Covering a distance of 600–700 m was considered satisfactory, while a distance of fewer than 300 m indicated a poor prognosis [24]. Before the test, the respondents were informed of the possibility of resting during the test in a standing or sitting position (on chairs located along the corridor) when symptoms of exercise intolerance (dyspnoea, shortness of breath, maximum pain of the lower extremities, general fatigue, etc.) intensified.

In the case of severe symptoms of exercise intolerance (dyspnoea, shortness of breath, dizziness, blurred vision, sudden sweating, cyanosis, general weakness, and fatigue) that did not subside despite a temporary rest in a sitting or standing position, the test was discontinued immediately.

The test results consisted of a distance, measured in m (with accuracy of 1 m), and a level of subjective fatigue according to the 10-point Borg scale (where 0 is no fatigue/dyspnoea and 10 is extreme fatigue/shortness of breath). Before beginning the test, immediately after it, and in the fifth minute of restitution, as well as at a time when the respondent was resting as a result of intensifying symptoms of exercise intolerance, pulse rate measurements were taken at the radial artery of the upper extremity without a fistula. In addition, blood pressure readings were taken in a sitting position [24].

### 2.4. Spiroergometry

The test was performed using a k4b2 cardiopulmonary ergospirometer by COSMED (Italy), in line with the protocol developed at Bartnet-Lab using the cycle-ergometer by Kettler. The measurement of gas exchange during the exercise test (spiroergometry) is a sophisticated measurement for assessing exercise capacity.

The exercise test was conducted in the form of a progressive test. During the first minute of the test, resting parameters were recorded. The initial power load for both women and men was 25 W. Patients pedalled on the ergometer with a frequency of revolutions of 60–90 rpm. In each subsequent minute, the load was increased by 5 W for women and 10 W for men. The test lasted until the patients refused to continue or until the pedalling frequency dropped below 59 rpm.

After the completion of the test, for another three minutes, the ergospirometer registered the restitution parameters. The following parameters were analysed: minute ventilation VE (L/min), oxygen uptake VO_2_ (mL/min), and oxygen uptake per kg of body weight VO_2_/kg (mL/min/kg). These were maximal values obtained in the progressive maximal test (largest values, averaged over 15-second intervals).

### 2.5. Tai Chi Training

The Tai Chi training was held twice a week for 60 minutes for a period of six months (48 training sessions). Training sessions took place at a gymnasium in the Department of Physiotherapy of the University of Physical Education in Wroclaw on days without dialysis, under constant supervision of a certified Tai Chi specialist and an attending practitioner. The Tai Chi 24-form Yang (24 forms of Beijing) was used in the training. The form 24 Tai Chi Chuan of the Yang style was the most common form of Tai Chi training, defined as a standardised form from Beijing [25].

The intensity of classes increased gradually. For patients' safety, a team of therapists were monitoring the limits permissible in cardiac rehabilitation concerning heart rate and blood pressure, as defined by international standards [26]. In addition, the basic parameter determining the maximum safe level of physical activity was the heart rate limit (HR_max_), which was determined individually for each patient during the exercise stress test. An increase in the heart rate of up to 50% of HR_max_ in weeks 1–4, up to 60% of the rate in weeks 4–8, up to 70% in weeks 8–16, and up to 80% in weeks 16–24 was deemed safe. An allowable subjective submaximal fatigue during training, not exceeding 7-8 points on a simplified 10-point Borg scale, was also considered.

### 2.6. Statistical Analysis

A statistical description of the data included the determination of mean values, standard deviations, and a range of variations, that is, maximum and minimum values in the distribution of a characteristic. The normality of the distribution of analysed characteristics was assessed using the Shapiro-Wilk test. The sexual dimorphism of the age distribution was evaluated using Student's *t*-test for independent samples. Changes in the distribution of characteristics as a result of the six-month Tai Chi training were examined using Student's *t*-test for dependent samples. Correlations were evaluated using Pearson's correlation coefficient (*r*). The critical significance level was set at *α* = 0.05. Statistical analysis was conducted using STATISTICA 10 software package by StatSoft.

## 3. Results

Twenty patients met the study criteria. At the end of the research project, 14 patients remained (Figure 1). Their mean age was 69.2 ± 8.6 years, including seven women and seven men. The characteristics of the study group are shown in Table 1.

The average distance achieved in the 6MWT test was 387.89 m, which improved significantly after six months of Tai Chi training (*P* = 0.019). There was also a decline in subjective fatigue as a result of physical effort, which was assessed using a 10-point Borg scale (*P* = 0.002) (Table 2). Mean values of heart rate frequency and blood pressure did not change significantly (Table 3).

The analysis of changes to the spiroergometric parameters in response to a six-month cycle of Tai Chi showed a statistically significant prolongation of the exercise stress test regarding time using a cycloergometer (*P* = 0.013). The remaining parameters were not statistically significant (Table 4).

The analysis of the correlation showed a positive relationship between the distance obtained in the 6MWT and VE, VO_2 _mL/min, and VO_2_/kg both prior to partaking in Tai Chi exercises (*t*_1_) and after six months of training (*t*_2_) (Table 5). The correlation of respondents' ages with the 6MWT test results and the spiroergometric variables was negative and statistically insignificant.

## 4. Discussion

Thus far, only a few scientific reports confirmed the beneficial effects of Tai Chi training among patients with ESRD. Positive effects were confirmed in studies on physical fitness, muscular strength, balance and falls, cardiorespiratory parameters, immune system efficacy, quality of sleep, and quality of life [27–32]. However, the subject of Tai Chi training in kidney diseases is still poorly understood.

The results obtained showed that, after six months of Tai Chi training conducted on days without dialysis, the average walking distance was lengthened by 12.5%. The improvement applied to all respondents and was statistically significant, as opposed to studies by Ling et al. [20], who evaluated, among others, walking capacity of ESRD patients using the 6MWT. The improvement recorded over the average walking distance in the 6MWT after three months of training was only 4.1%. The reason could regard the unsupervised nature of the proposed exercises (at home using a video), as well as a shorter, three-month period of training [20].

The subjective feeling of fatigue following exercise was reduced as a result of long-term, systematic Tai Chi training. Prior to the six-month programme, the physical effort exerted during the test was perceived as very hard; after Tai Chi training, it was perceived as somewhat hard. This additionally showed an increase in exercise tolerance among respondents. This was also confirmed in the correlation analysis, which indicated a significant relationship between distance covered in the 6MWT test and exercise capacity. Therefore, the longer the distance a patient covered, the greater the exercise capacity. This was important for patients with ESRD and it was reflected in the improvement in their quality of life and daily functioning.

The evaluation of exercise capacity by means of spiroergometry showed a significant increase in exercise stress test time (by 1.37 minutes). The respiratory parameters examined did not change significantly. This was probably because Tai Chi training was a low-intensity exercise, which did not significantly increase respiratory rate; thus, it only slightly improved the respiratory component of exercise capacity. Therefore, the main process of the body's physiological adaptation to physical activity must take place in the cardiovascular and muscular system.

Thus far, the effects of Tai Chi training on the level of exercise capacity in dialysis patients were not analysed. However, many studies conducted among people with cardiovascular diseases, which often coexist in patients with ESRD, confirmed the significant impact of long-term (over three months) Tai Chi training on aerobic capacity, especially in patients that had previously functioned at a very low level of physical activity [28, 33].

It was worth noting that, in patients with ESRD, more benefits were obtained when the training was performed on days without dialysis, though it was burdened with a higher percentage of resignation among participants (up to 50%) [20]. Furthermore, Konstantinidou et al. (2002) confirmed a greater effectiveness in improving exercise capacity among people training on days without dialysis compared to training performed during dialysis [16]. Also, in the present study, Tai Chi training conducted on days without dialysis produced measurable benefits. This method of rehabilitation seemed to be more attractive and encouraging compared to training during dialysis, while the group character of the activities additionally motivated people to continue this form of training, thus improving the psychophysical condition of patients.

The obtained results require further research and verification on a larger group of patients with ESRD. A control group should also be introduced, which is a limitation of this study. Nevertheless, even at this stage, it can be said that Tai Chi training for six months significantly improves exercise capacity and walking qualities and can be introduced into the rehabilitation programmes of dialysis patients due to its safe and effective form.

In conclusion, after six months of Tai Chi training, the average 6MWT score and the time on a cycle-ergometer significantly improved, which indicated a definite improvement in exercise tolerance in all respondents. Tai Chi training conducted on days without dialysis was an effective and interesting form of rehabilitation for patients, providing a chance for fewer falls and hospitalisations and a higher quality of life.

## Figures and Tables

**Table 1:** Baseline characteristics of the patients.

Baseline characteristics	Total patients (%) (*n* = 14)
Age	
Mean (SD)	69.2 (8.6)
Range	55–83

Gender	
Women	7 (50)
Men	7 (50)

Education	
Primary and vocational	2 (14.3)
Secondary	8 (57.14)
Higher	4 (28.57)

Marital status	
Married	7 (50)
Single people (widow(er), unmarried)	7 (50)

Cause of ESRD:	
Nephropathy (diabetic + hypertensive)	6 (42.85)
Glomerulonephritis	5 (35.72)
ADPDK	2 (14.3)
Other	1 (7.14)

Disease duration (years)	
Mean (SD)	11.5 (9.2)

Duration of dialysis (years)	
Mean	7.2 (4.1)

Number of comorbidities	
Mean (SD)	2.85 (2.1)

Comorbidities:	
Hypertension	11
Diabetes	5
Cardiovascular disease (ischemic heart disease, atrial fibrillation, TIA, stroke)	9
Peripheral artery disease	7
Degenerative joint disease	2
Other	6

**Table 2:** The changes in result of 6MWT after 6 months of Tai Chi training.

Variable	Measurement	Mean	SD	Mean change	Student's *t*-test	
					*t*	*P*
6MWT [m]	*t*_1_	387.89	139.80	48.5	2.67	0.019^a^
	*t*_2_	436.36	186.49			

Borg scale	*t*_1_	7.4	0.7	2.7	8.80	0.002^a^
	*t*_2_	4.7	0.7			

^a^ *P* < 0.05; *t*_1_: before Tai Chi training; *t*_2_: after Tai Chi training.

**Table 3:** Effects of Tai Chi training on heart rate and blood pressure before and after 6MWT.

Variable	Measurement	Mean		Mean change	Student's *t*-test	
		Before 6MWT	After 6MWT		*t*	*P*
HR, bpm	*t*_1_	79.1	89.3	10.2	0.18	0.857
	*t*_2_	78.7	88.4	9.6		

SBP, mmHg	*t*_1_	125.6	151.0	25.4	1.20	0.253
	*t*_2_	133.4	149.4	15.9		

DBP, mmHg	*t*_1_	74.9	85.5	10.6	1.19	0.257
	*t*_2_	77.9	84.6	6.7		

^a^ *P* < 0.05; DBP: diastolic blood pressure; HR: heart rate; SBP: systolic blood pressure; *t*_1_: before Tai Chi training; *t*_2_: after Tai Chi training.

**Table 4:** Changes of spiroergometry parameters after 6-month Tai Chi training.

Variable	Measurement	Mean	SD	Mean change	Student's *t*-test	
					*t*	*P*
Test time, min	*t*_1_	8.71	2.76	1.37	2.89	0.013^a^
	*t*_2_	10.08	3.72			

MET	*t*_1_	4.34	1.53	0.35	1.72	0.109
	*t*_2_	4.69	1.90			

VE, L/min	*t*_1_	43.00	16.98	0.89	0.55	0.594
	*t*_2_	43.89	18.17			

VO_2_, mL/min	*t*_1_	1096.6	502.9	96.00	1.70	0.113
	*t*_2_	1192.6	609.8			

VO_2_/kg, mL/min/kg	*t*_1_	15.37	5.55	0.98	1.64	0.125
	*t*_2_	16.36	6.48			

^a^ *P* < 0.05; *t*_1_: before Tai Chi exercise training; *t*_2_: after Tai Chi exercise training; VE: minute ventilation; VO_2_: oxygen uptake; VO_2_/kg: oxygen uptake per kg of body weight.

**Table 5:** Pearson's correlation of 6MWT with spiroergometry parameters before and after Tai Chi training.

6MWT	VE, L/min	VO_2_, mL/min	VO_2_/kg, mL/min/kg
*t*_1_	0.83^a^	0.78^a^	0.77^a^
*t*_2_	0.87^a^	0.78^a^	0.83^a^

^a^Significant Pearson's correlation; *r*_*k*_ (0.05) = 0.53; *t*_1_: before Tai Chi training; *t*_2_: after Tai Chi training; VE: minute ventilation; VO_2_: oxygen uptake; VO_2_/kg: oxygen uptake per kg of body weight.

## References

[1] Johansen K. L., Kaysen G. A., Young B. S., Hung A. M., da Silva M., Chertow G. M. (2003). Longitudinal study of nutritional status, body composition, and physical function in hemodialysis patients. *The American Journal of Clinical Nutrition*.

[2] McIntyre C. W., Selby N. M., Sigrist M., Pearce L. E., Mercer T. H., Naish P. F. (2006). Patients receiving maintenance dialysis have more severe functionally significant skeletal muscle wasting than patients with dialysis-independent chronic kidney disease. *Nephrology Dialysis Transplantation*.

[3] Johansen K. L., Doyle J., Sakkas G. K., Kent-Braun J. A. (2005). Neural and metabolic mechanisms of excessive muscle fatigue in maintenance hemodialysis patients. *American Journal of Physiology—Regulatory Integrative and Comparative Physiology*.

[4] Painter P., Messer-Rehak D., Hanson P., Zimmerman S. W., Glass N. R. (1985). Exercise capacity in hemodialysis, CAPD and renal transplant patients. *Nephron*.

[5] Johansen K. L., Chertow G. M., Ng A. V. (2000). Physical activity levels in patients on hemodialysis and healthy sedentary controls. *Kidney International*.

[6] Inker L. A., Astor B. C., Fox C. H. (2014). KDOQI US commentary on the 2012 KDIGO clinical practice guideline for the evaluation and management of CKD. *American Journal of Kidney Diseases*.

[7] Cheema B. S. B., Fiatarone Singh M. A. (2005). Exercise training in patients receiving maintenance hemodialysis: a systematic review of clinical trials. *American Journal of Nephrology*.

[8] Chojak-Fijałka K., Smolenski O. (2006). Rehabilitacja ruchowa chorych przewlekle hemodializowanych—wyniki badań własnych. *Problemy Lekarskie*.

[9] Gołębiowski T., Kusztal M., Weyde W. (2012). A program of physical rehabilitation during hemodialysis sessions improves the fitness of dialysis patients. *Kidney and Blood Pressure Research*.

[10] Girija K., Radha R. (2013). Beneficial effect of physical activity in hemodialysis patients. *Universal Journal of Engineering Science*.

[11] Dziubek W., Bulińska K., Rogowski Ł. (2015). The effects of aquatic exercises on physical fitness and muscle function in dialysis patients. *BioMed Research International*.

[12] Kouidi E. J. (2001). Central and peripheral adaptations to physical training in patients with end-stage renal disease. *Sports Medicine*.

[13] Kouidi E., Albani M., Natsis K. (1998). The effects of exercise training on muscle atrophy in haemodialysis patients. *Nephrology Dialysis Transplantation*.

[14] Storer T. W., Casaburi R., Sawelson S., Kopple J. D. (2005). Endurance exercise training during haemodialysis improves strength, power, fatigability and physical performance in maintenance haemodialysis patients. *Nephrology Dialysis Transplantation*.

[15] Painter P., Carlson L., Carey S., Paul S. M., Myll J. (2000). Physical functioning and health-related quality-of-life changes with exercise training in hemodialysis patients. *American Journal of Kidney Diseases*.

[16] Konstantinidou E., Koukouvou G., Kouidi E., Deligiannis A., Tourkantonis A. (2002). Exercise training in patients with end-stage renal disease on hemodialysis: comparison of three rehabilitation programs. *Journal of Rehabilitation Medicine*.

[17] Strath S. J., Kaminsky L. A., Ainsworth B. E. (2013). Guide to the assessment of physical activity: clinical and research applications: a scientific statement from the American Heart Association. *Circulation*.

[18] Jamal S. A., West S. L., Miller P. D. (2012). Fracture risk assessment in patients with chronic kidney disease. *Osteoporosis International*.

[19] Bonifonte S. P. (2004). *Tai Chi for Seniors*.

[20] Ling K.-W., Wong F. S. Y., Chan W.-K. (2003). Effect of a home exercise program based on Tai Chi in patients with end-stage renal disease. *Peritoneal Dialysis International*.

[21] Shahgholian N., Eshghinezhad A., Mortazavi M. (2014). The effect of tai chi exercise on quality of life in hemodialysis patients. *Iranian Journal of Nursing and Midwifery Research*.

[22] Mustafa S., Cooper L., Langrick N., Simon N., Jassal S. V., Oreopoulos D. G. (2005). The effect of a Tai Chi exercise program on quality of life in patients on peritoneal dialysis: a pilot study. *Peritoneal Dialysis International*.

[23] Phisitkul S., Chyu M. C., Zhang Y. (2013). Intradialytic modified Tai Chi exercise among end-stage renal disease patients undergoing hemodialysis: an exploratory pilot study. *Alternative & Integrative Medicine*.

[24] ATS Committee on Proficiency Standards for Clinical Pulmonary Function Laboratories (2002). ATS statement: guidelines for the six-minute walk test. *American Journal of Respiratory and Critical Care Medicine*.

[25] Topor A., Kajfasz A., Chuan T. C. (2006). *The Path of Health, Longevity and Martial Arts. Part I*.

[26] Dylewicz P., Jegier A., Piotrowicz R. (2004). Kompleksowa rehabilitacja kardiologiczna. *Folia Cardiologica*.

[27] Wang C., Collet J. P., Lau J. (2004). The effect of Tai Chi on health outcomes in patients with chronic conditions: a systematic review. *Archives of Internal Medicine*.

[28] Verhagen A. P., Immink M., van der Meulen A., Bierma-Zeinstra S. M. A. (2004). The efficacy of Tai Chi Chuan in older adults: a systematic review. *Family Practice*.

[29] Kuramoto A. M. (2006). Therapeutic benefits of Tai Chi exercise: research review. *Wisconsin Medical Journal*.

[30] Yeh G. Y., Wang C., Wayne P. M., Phillips R. S. (2008). The effect of Tai Chi exercise on blood pressure: a systematic review. *Preventive Cardiology*.

[31] Jahnke R., Larkey L., Rogers C., Etnier J., Lin F. (2010). A comprehensive review of health benefits of qigong and Tai Chi. *American Journal of Health Promotion*.

[32] Wang C., Bannuru R., Ramel J., Kupelnick B., Scott T., Schmid C. H. (2010). Tai Chi on psychological well-being: Systematic review and meta-analysis. *BMC Complementary and Alternative Medicine*.

[33] Taylor-Piliae R. E., Hong Y. (2008). The effectiveness of Tai Chi exercise in improving aerobic capacity: an updated meta-analysis. *Tai Chi Chuan. State of the Art in International Research*.

